# District-Level Inequalities in Hypertension among Adults in Indonesia: A Cross-Sectional Analysis by Sex and Age Group

**DOI:** 10.3390/ijerph192013268

**Published:** 2022-10-14

**Authors:** Puput Oktamianti, Dian Kusuma, Vilda Amir, Dwi Hapsari Tjandrarini, Astridya Paramita

**Affiliations:** 1 Health Administration and Policy Department, Faculty of Public Health, Universitas Indonesia, Depok 16424, Indonesia; 2 Department of Health Services Research and Management, School of Health & Psychological Sciences, City University of London, London EC1V 0HB, UK; 3 Center for Health Administration and Policy Studies, Faculty of Public Health, Universitas Indonesia, Depok 16424, Indonesia; 4 Research Center for Public Health and Nutrition, National Research and Innovation Agency, Bogor 16915, Indonesia

**Keywords:** high blood pressure, disparity, geographic, socioeconomic, Indonesia

## Abstract

Background: An estimated 1.28 billion adults 30–79 years old had hypertension globally in 2021, of which two-thirds lived in low- and middle-income countries (LMICs). Previous studies on geographic and socioeconomic inequalities in hypertension among adults have limitations: (a) most studies used individual-level data, while evidence from locality-level data is also crucial for policymaking; (b) studies from LMICs are limited. Thus, our study examines geographic and socioeconomic inequalities in hypertension among adults across districts in Indonesia. Methods: We combined geospatial and quantitative analyses to assess the inequalities in hypertension across 514 districts in Indonesia. Hypertension data were obtained from the Indonesian Basic Health Survey (Riskesdas) 2018. Socioeconomic data were obtained from the World Bank. Six dependent variables included hypertension prevalence among all adults (18+ years), male adults, female adults, young adults (18–24 years), adults (25–59 years), and older adults (60+ years). Results: We also found significant geographic and socioeconomic inequalities in hypertension among adults across 514 districts. All hypertension indicators were higher in the most developed region than in the least developed region. Districts in the Java region had up to 50% higher prevalence of hypertension among all adults, males, females, young adults, adults, and older adults. Notably, districts in the Kalimantan region had the highest prevalence of hypertension, even compared to those in Java. Moreover, income level was positively associated with hypertension; the wealthiest districts had higher hypertension than the poorest districts by up to 30%, but only among males and older adults were statistically significant. Conclusions: There were significant inequalities in hypertension among adults across 514 districts in the country. Policies to reduce such inequalities may need to prioritize more affluent urban areas and rural areas with a higher burden.

## 1. Background

The World Health Organization (WHO) estimated that 1.28 billion adults 30–79 years old had hypertension globally in 2021, of which two-thirds lived in low-and middle-income countries (LMICs) [1]. It is a serious medical condition of elevated blood pressure that increases the risks of diseases such as heart, brain, and kidney [1]. The latest Global Burden of Diseases study found that high blood pressure was the top leading risk of death and disability among adults in 2019 [2], which may contribute to ischemic heart disease, stroke, and chronic kidney diseases being among the top ten leading causes of deaths and disability in the same year [3]. Moreover, the economic burden is substantial. A recent study from Ethiopia showed total productivity loss due to premature mortality and morbidity was over USD 449,000, and the overall economic burden of hypertension was over USD 514,000 (or USD 106 per person per month) [4].

Indonesia is the fourth most populated country, with over 276 million people in 2021. Like many LMICs, Indonesia is a lower-middle-income country with an increasing burden of hypertension. The nationally representative surveys of the Indonesia Basic Health Survey (Riskesdas) found that hypertension among adults 18+ years old increased rapidly from 25.8% in 2013 to 34.1% in 2018 [5]. The latest national-level Global Burden of Study found that high blood pressure was the top risk factor attributable to deaths and disabilities in Indonesia, which may contribute to ischemic heart disease and cerebrovascular disease being the first and second leading cases of deaths and disabilities in the country [6].

The relationships between socioeconomic indicators and hypertension among adults have been well-studied, including in LMICs. Busingye et al. [7] conducted a meta-analysis and found that overall, there was a positive association between hypertension and income, while no association with educational status. However, they found that educational status was inversely associated with hypertension in East Asia but positively associated in South Asia. Mishra et al. assessed the socioeconomic inequalities using Nepal Demographic Health Survey data and found that adults from the highest education and income groups were 1.4 times and 1.7 times more likely to be hypertensive than those from the lowest education and income groups [8]. Previous studies have also shown some evidence of geographic inequalities in adult hypertension. Kershaw et al. [9] analyzed participants from six study sites in the United States and found that Blacks born in southern states were 1.11 times more likely to be hypertensive than non-southern states (findings were not significant for whites). Morenoff et al. [10] analyzed the Chicago Community Adult Health Study and found that hypertension was negatively associated with neighborhood affluence. Cho et al. [11] analyzed data from Korean National Health Insurance and found that neighborhood deprivation can exacerbate the influence of individual SES on all-cause mortality among patients with newly diagnosed hypertension.

Effective responses to reduce the inequalities in hypertension are crucial to achieving one of the global targets for non-communicable diseases—to reduce the prevalence of hypertension by 33% between 2010 and 2030 [1]. However, previous studies on geographic and socioeconomic inequalities in hypertension among adults have at least two limitations. First, the majority used individual-level data, including studies from Asia, Africa, and Latin America [7,8]. While such studies are essential, evidence from locality-level data (such as districts) is also crucial for policymaking, especially in a decentralized setting such as Indonesia, where some policies are transferred to the district level. Second, previous studies on geographic inequalities are mainly from high-income countries such as the United States and South Korea [9,10,11]. Studies from LMICs such as China and Thailand are limited to analysis using urban/rural or provincial levels [12,13,14]. Thus, our study aims to examine geographic and socioeconomic inequalities in hypertension among adults across 514 districts in Indonesia.

## 2. Methods

### 2.1. Study Design

Using a cross-sectional study, we analyzed geographic and socioeconomic disparities in hypertension among adults aged 18+ years in Indonesia. Geographic disparities were analyzed using geospatial analyses across 34 provinces and 514 districts. Socioeconomic disparities were assessed using multivariate regression analyses across 514 districts. Hypertension data as the primary dependent variable were obtained from the latest RISKESDAS 2018, a nationally representative health survey. The survey collected information on maternal and child health, nutrition status, communicable and non-communicable diseases and main risk factors, health behaviors, and disability among children and adults [5]. In total, the survey targeted 300,000 households using two-stage sampling. First, the team selected 30,000 census blocks in each urban and rural using probability proportional to size out of a total of 720,000 census blocks in the country. Second, ten households were systematically chosen using implicit stratification of the household head’s education. For adults, the survey included 624,563 individuals aged 18+ years [5].

### 2.2. Independent Variables

The main independent variables included region, urban/rural, income, and education level at the district level, obtained from the World Bank database. For the region, we divided provinces and districts into five: Sumatera, Java (including Bali), Kalimantan, Sulawesi, and Papua (including Nusa Tenggara and Maluku). A reference to the provinces and regions is provided in Appendix A. In Indonesia, the western part is generally more developed (especially Java and Bali) than the eastern part (including Papua, Nusa Tenggara, and Maluku) [15,16,17]. In terms of urban and rural, we conducted the analyses using all districts, urban districts (i.e., cities) and rural districts (i.e., regencies). By income level, we grouped district-level poverty rates into five quintiles, with quintile one being the poorest (or highest poverty rates) and quintile five being the wealthiest (or lowest poverty rates). By education level, we grouped the net enrollment ratios of senior secondary into five quintiles, with quintile 1 being the least educated and quintile 5 being the most educated [15,16,17].

### 2.3. Dependent Variables

We used six indicators of hypertension as dependent variables: hypertension among all adults aged 18+ years, male adults, female adults, young adults aged 18–24 years, adults aged 25–59 years, and older adults aged 60+ years. Hypertension was defined as either systolic blood pressure 140+ mmHg, diastolic blood pressure 90+ mmHg, or both. A digital blood pressure monitor was used with respondents in a sitting position. Only two measurements were taken if the difference in blood pressure was less than 10 mmHg; otherwise, three were taken. For each participant, the average (mean) blood pressure was calculated from two measurements with the least difference. We assessed the prevalence by sex to observe variations for males and females. We evaluated the prevalence by age category to observe variations among young adults, adults, and older adults, which is crucial for better targeting NCD control and prevention efforts, including reforms toward effective health systems in Indonesia and other LMICs [18].

### 2.4. Data Analysis

For geospatial analyses, we divided the prevalence of hypertension among 34 provinces and 514 districts by quintile using ArcMap 10. For multivariate regression analysis, we performed Ordinary Least Square (OLS) models using STATA 15 to examine the associations between geographic indicators such as urban/rural and region and between socioeconomic indicators such as income and education level and each hypertension indicator: hypertension among all adults, male adults, female adults, young adults, adults, and older adults. We also calculated absolute and relative differences for the geographic and socioeconomic variations. We compared the differences between the most developed (the Java region) and the least developed region (the Papua region). We compared the differences between quintile 1 (poorest or least educated) and quintile 5 (wealthiest or most educated). All statistical significance was at the 5% level or lower.

## 3. Results

### 3.1. Provincial-Level Results

**Figure 1:**
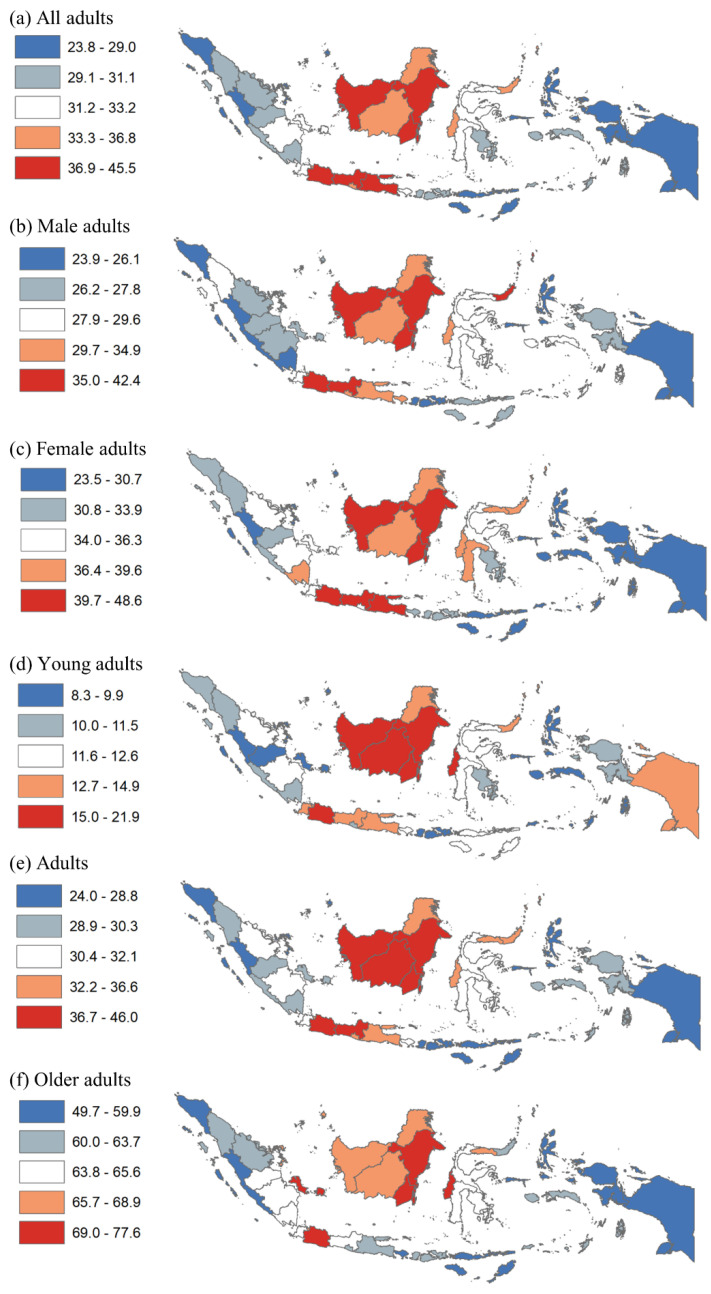
Disparity of hypertension among adults by province in Indonesia, 2018. Note: Numbers show the prevalence of hypertension among all adults, males, females, young adults, adults, and older adults.

Figure 1 shows the prevalence of hypertension among adults by quintile at the province level. In panels a–f, hypertension among all adults ranged from 23.8% to 45.5%; that among male adults ranged from 23.9% to 42.4%; that among female adults ranged from 23.5% to 48.6%; that among young adults ranged from 8.3% to 21.9%; that among adults ranged from 24.0% to 46.0%; that among older adults ranged from 49.7% to 77.6%. Among all adults, hypertension was highest (quintiles 4–5) in all provinces in Kalimantan, most provinces in Java (except for Banten province), and some in Sulawesi (e.g., North Sulawesi and West Sulawesi). In Kalimantan, this patterning was similar in other indicators, including hypertension among males, females, young adults, adults, and older adults. In Java, the patterning was similar in all other indicators except among older adults, with only West Java having the highest prevalence. By sex, additional provinces with the highest prevalence (quintiles 4–5) include Bali for males and Lampung and South Sulawesi for females. By age group, additional provinces with the highest prevalence (quintiles 4–5) include Banten and Papua for young adults, Gorontalo for adults, and Riau Islands, Bangka Belitung, and Gorontalo for older adults.

Table 1 shows the prevalence of hypertension among adults by province. The top and bottom boxes show the ten wealthiest and poorest provinces, respectively. The grey-shaded cells show a prevalence higher than the national average for each column of the hypertension indicator. Five of the ten wealthiest provinces (including South Kalimantan, Central Kalimantan, North Kalimantan, East Kalimantan, and Jakarta) had consistently higher than average for at least five indicators. In contrast, none of the ten poorest provinces did.

### 3.2. District-Level Results

Table 2 shows the descriptive statistics of districts in our analysis, including the prevalence of hypertension among adults. Of 514 districts, 97 (18.9%) were urban cities, and 417 (81.1%) were rural regencies. Urban cities were mainly in Java (36.1% of 97) and Sumatera (34.0%). Rural regencies were less concentrated, including 29.0% (of 417 regencies) in Java, 22.3% in Sumatera, 20.6% in Papua, 16.8% in Sulawesi, and 11.3% in Kalimantan). By the level of income, 79% of urban areas were wealthier (quintiles 4–5), while nearly half (47.2%) of rural areas were poorer (quintiles 1–2). By the level of education, 71.1% of urban cities had higher education (quintiles 4–5), while nearly half (46.8%) of rural regencies had lower education (quintiles 1–2). Regarding the dependent variables, the prevalence of hypertension was 33.3% among all adults, 30.4% and 36.0% among males and females, and 12.9%, 32.6%, and 63.2% among young adults, adults, and older adults, respectively. Compared to rural areas, hypertension among males, adults, and older adults was significantly higher in urban areas but significantly lower among females. Hypertension among males, adults, and older adults was 32.6%, 34.0, and 66.2% in urban areas and 29.9%, 32.3%, and 62.5% in rural areas. Hypertension among females was 34.6% and 36.4% in urban and rural areas.

**Figure 2:**
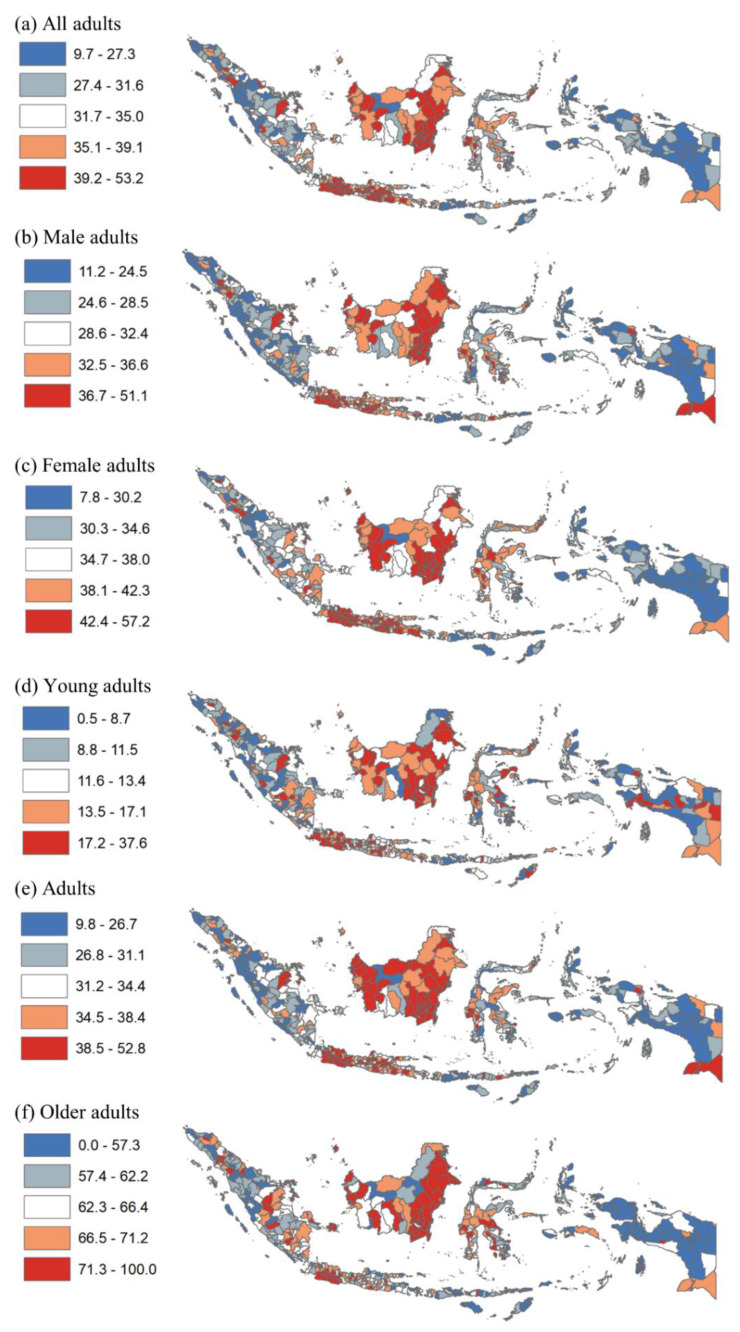
Disparity of hypertension among adults by district in Indonesia, 2018. Note: Numbers show prevalence of hypertension among all adults, males, females, young adults, adults, and older adults.

Figure 2 shows the prevalence of hypertension by quintile at the district level, showing more granularity than at the provincial level. For instance, many districts in Aceh, North Sumatera, Riau, South Sumatera, Lampung, Bali, East Nusa Tenggara, West Nusa Tenggara, Central Sulawesi, Southeast Sulawesi, and Papua provinces had the highest prevalence of hypertension (quintiles 4–5) among all adults. In contrast, several districts in West Kalimantan and Central Kalimantan had a lower prevalence of hypertension (quintiles 1–2). This patterning is similar for hypertension among males, females, young adults, adults, and older adults.

In terms of socioeconomic disparities, Appendix C and Appendix D provide ten districts with the lowest and highest prevalence of hypertension among adults, respectively. For all adults, the prevalence of hypertension ranged from 9.7% in Nduga regency (Papua province) to 53.2% in Hulu Sungai Tengah (Papua). By sex, hypertension among males ranged from 11.0% in Nduga (Papua) to 51.1% in Kutai Barat (East Kalimantan); hypertension among females ranged from 8.0% in Nduga (Papua) to 57.2% in Ciamis (West Java). By age group, hypertension among young adults ranged from 1% in Buton Tengah (Southeast Sulawesi) and Mentawai Islands (West Sumatera) to 37.6% in Pegunungan Bintan Yalimo (Papua); that among adults ranged from 9.8% in Nduga (Papua) to 52.8% in Kutai Barat (East Kalimantan); that among older adults ranged from 0% in Yahukimo, Pegunungan Bintan, and Nduga (Papua) to 100% in Diyai (Papua). By urban/rural, all districts with the lowest prevalence of hypertension for all adults, by sex, and by age groups were rural. Similarly, most districts with the highest prevalence of hypertension for all adults by sex and age groups were rural. By income, the average poverty rates among the ten districts with the highest prevalence of hypertension were up to 14%, while the rates among the districts with the lowest prevalence were up to 35%.

Table 3 shows the associations between geographic and socioeconomic indicators (i.e., region, income, and education) and hypertension. The absolute (relative) values indicate the difference (ratio) between the most (Java and Bali) vs. the least (Papua, Nusa Tenggara, and Maluku) developed regions, the wealthiest (quintile 5) and poorest (quintile 1) districts, and the most educated (quintile 5) and least educated (quintile 1) districts. By region, districts in the most developed region had a significantly higher prevalence of hypertension among all adults, males, females, young adults, adults, and older adults, compared to those in the least developed region. Districts in Java had 45%, 40%, 50%, 29%, 40%, and 27% higher prevalence of hypertension among all adults, males, females, young adults, adults, and older adults, respectively. However, districts in the Kalimantan region had the highest prevalence of hypertension among all adults, by sex, and by age group, compared to districts in all other regions, including Java. By income, the wealthiest districts had a higher prevalence of hypertension among all adults, by sex, and by age group than the poorest districts. However, only hypertension among males and older adults was statistically significant—the wealthiest districts had a 30% and 24% higher prevalence among males and older adults. By education, the associations were mixed but mostly not significant except for hypertension among young adults, which was significantly higher in the least educated districts compared to the most educated ones. The least educated districts had a 22.0% (i.e., 1/0.82 = 1.22) higher prevalence of hypertension among young adults. Results were similar in the urban and rural subgroup analyses.

## 4. Discussion

We found a high prevalence of hypertension among adults 18+ years in Indonesia in 2018. The prevalence of hypertension was 33.3%, 30.4%, and 36.0% among all adults, males, and females, respectively. By age, the prevalence was 12.9%, 32.6%, and 63.2% among young adults (18–24 years), adults (25–59 years), and older adults (60 years and over), respectively. The findings are similar to the global the global estimates of age-standardized hypertension prevalence in adults 30–79 years of 32% in women and 34% in men in 2019 [19].

We also found a significant geographic and socioeconomic disparity in hypertension among adults across 514 districts in Indonesia. By urbanicity, while overall hypertension was generally higher in urban areas in Indonesia, we found mixed results by sex. Hypertension among males was significantly higher in urban areas (32.6% in urban vs. 29.9% in rural), but that among females was higher in rural areas (34.6% in urban vs. 36.4% in rural). This evidence aligns with a study in Turkey that found that women were more likely to be hypertensive in rural areas than in urban areas [20]. However, other studies from Nepal and Ghana found that hypertension among female adults was higher in urban areas [8,21]. Moreover, at the district level, while all districts with the lowest hypertension for all adults, by sex, and by age groups were rural, many districts with the highest prevalence were also rural. Thus, effective responses to reduce disparity in hypertension may need to prioritize not only urban areas but also rural areas with an already high burden of hypertension [22,23,24].

By region, all hypertension indicators were higher in the most developed region (i.e., the Java region, including Bali) than in the least developed region (e.g., the Papua region, including Maluku and Nusa Tenggara). Similarly, by income, the wealthiest districts had higher hypertension among all adults, by sex, and by age group than the poorest districts (although only among males and older adults was statistically significant). All this finding aligns with previous studies from LMICs. Studies on geographic variations across 31 provinces in China found that hypertension was higher in more developed areas such (e.g., Beijing and Shanghai) than in less developed areas such as (e.g., Hainan) [12,13]. In addition, a study across 76 provinces in Thailand found that hypertension was higher in Bangkok and metropolitan areas and lower in the northeast and southern provinces [14]. In contrast, studies from high-income countries such as the United States and South Korea found that hypertension was higher among less developed areas or neighborhoods [9,10,11].

For policy, hypertension is increasing among young adults and is already high among the adult population in the country, which is likely to produce a substantial economic burden from total productivity loss due to premature mortality and morbidity [4]. Also, the hypertension burden among older adults is very high. All this indicates the need for health systems reform towards improved prevention of non-communicable diseases and their main risk factors, especially hypertension. Reforms may include changes from the community to primary care and secondary care and integration with infectious disease platforms [25,26,27]. By region and socioeconomic status, effective responses to reduce inequalities in hypertension may need to prioritize more affluent urban areas and rural areas with higher hypertension burden and other risk factors for non-communicable diseases [28,29,30,31,32,33].

To the best of our knowledge, our study is the first in LMICs to examine geographic and socioeconomic inequalities in hypertension among all adults, males, females, young adults, adults, and older adults across many local units (over 500 districts). However, our study also has at least two limitations. First, we did not have information on ethnicity in our dataset, which limits our sub-group analysis by that variable [34,35]. Secondly, we used cross-sectional data and could not assess trends over time. Despite these limitations, our findings are highly relevant to health policies in Indonesia and other LMICs.

## 5. Conclusions

In Indonesia, hypertension prevalence was highest among females (36.0%) and older adults 60+ years (63.2%). We found significant geographic and socioeconomic inequalities in the prevalence of hypertension among adults across 514 districts. Hypertension was higher in the most developed region than in the least developed region. Districts in the Java region had up to 50% higher prevalence of hypertension among all adults, males, females, young adults, adults, and older adults. Notably, districts in the Kalimantan region had the highest prevalence of hypertension, even compared to those in Java. Moreover, income level was positively associated with hypertension; the wealthiest districts had higher hypertension than the poorest districts by up to 30%, but only among males, and older adults were statistically significant. Policies to reduce such inequalities may need to prioritize more affluent urban districts and rural areas with a higher burden.

## Figures and Tables

**Table 1:** Prevalence of hypertension among adults by province in Indonesia, 2018.

		Hypertension Prevalence					
	Poverty				Young		
	Rates	All	Males	Females	Adults	Adults	Older Adults
	(1)	(2)	(3)	(4)	(5)	(6)	(7)
Bali	4.5%	32.0%	32.8%	31.1%	12.3%	30.7%	56.4%
South Kalimantan	4.8%	45.5%	42.4%	48.6%	21.9%	46.0%	77.6%
Central Kalimantan	5.0%	35.9%	32.6%	39.6%	15.3%	36.7%	68.9%
Jakarta	5.0%	35.4%	34.9%	36.0%	12.7%	35.1%	68.9%
Banten	5.3%	31.4%	28.3%	34.5%	13.3%	31.6%	64.7%
Bangka Belitung	5.4%	31.5%	27.7%	35.6%	9.8%	30.3%	71.5%
West Sumatera	6.6%	27.1%	23.9%	30.0%	9.1%	25.2%	56.2%
North Kalimantan	7.0%	35.3%	33.7%	37.1%	12.7%	36.6%	65.8%
East Kalimantan	7.1%	41.2%	40.0%	42.6%	17.8%	42.3%	75.6%
Riau Islands	7.6%	28.1%	27.5%	28.8%	8.3%	28.3%	67.8%
Jambi	7.8%	30.1%	26.6%	33.7%	9.1%	29.6%	64.0%
North Maluku	7.9%	26.5%	24.3%	28.7%	9.5%	26.1%	59.2%
West Java	7.9%	40.9%	36.8%	45.0%	17.0%	40.6%	73.1%
West Kalimantan	8.1%	38.4%	36.1%	40.7%	16.0%	39.0%	67.5%
North Sulawesi	8.5%	36.8%	35.0%	38.7%	14.2%	35.9%	63.7%
Riau	8.8%	31.0%	27.8%	34.4%	12.5%	31.7%	63.2%
South Sulawesi	9.8%	33.2%	29.4%	36.7%	12.4%	32.1%	65.6%
West Sulawesi	10.3%	36.3%	33.7%	38.8%	15.2%	36.5%	70.7%
East Java	10.9%	37.7%	33.8%	41.3%	13.2%	36.4%	63.6%
Central Java	10.9%	38.8%	35.7%	41.7%	14.9%	36.9%	65.6%
North Sumatera	11.3%	30.3%	28.5%	32.1%	11.0%	29.7%	63.6%
Lampung	12.6%	31.1%	26.1%	36.4%	10.0%	30.0%	64.0%
Jogyakarta	12.7%	35.2%	34.1%	36.3%	11.5%	32.7%	62.9%
Southeast Sulawesi	13.0%	31.1%	29.6%	32.6%	11.2%	31.1%	64.7%
South Sumatera	13.1%	31.7%	27.8%	35.7%	12.1%	30.9%	65.3%
Central Sulawesi	14.6%	32.2%	28.5%	36.1%	12.0%	31.6%	64.1%
West Nusa Tenggara	14.8%	29.3%	24.5%	33.6%	8.6%	28.4%	63.4%
Bengkulu	15.0%	29.8%	25.9%	33.9%	10.5%	29.6%	59.9%
Aceh	16.4%	28.8%	25.2%	32.3%	10.8%	28.8%	59.9%
Gorontalo	16.8%	32.7%	28.2%	37.1%	12.6%	32.3%	68.8%
Maluku	21.8%	30.0%	29.2%	30.7%	9.9%	29.9%	63.3%
East Nusa Tenggara	22.0%	29.0%	27.3%	30.5%	11.8%	28.6%	54.7%
West Papua	26.5%	28.0%	27.7%	28.4%	11.1%	29.7%	53.8%
Papua	29.4%	23.8%	24.0%	23.5%	13.7%	24.0%	49.7%
AVERAGE		32.8%	30.3%	35.4%	12.5%	32.5%	64.3%

Note: Ordered by the average poverty rates (column 1), the provinces in the top box are the richest and those in the bottom box are the poorest. Shaded values show higher than the national average for each group.

**Table 2:** Characteristics of districts and hypertension among adults.

	All		Urban		Rural		Difference	
	n	%	n	%	n	%	%	
	(1)	(2)	(3)	(4)	(5)	(6)	(7) = (4–6)	
(a) Characteristics (#)								
Sample size district	514	100%	97	100%	417	100%	0%	
Region								
Papua	95	18.5%	9	9.3%	86	20.6%	11.3%	
Java	128	24.9%	35	36.1%	93	22.3%	−13.8%	
Sumatera	154	30.0%	33	34.0%	121	29.0%	−5.0%	
Kalimantan	56	10.9%	9	9.3%	47	11.3%	2.0%	
Sulawesi	81	15.8%	11	11.3%	70	16.8%	5.4%	
	514		97		417			
Income/poverty								
Q1 poor	102	19.8%	3	3.1%	99	23.7%	20.6%	
Q2	103	20.0%	5	5.2%	98	23.5%	18.3%	
Q3	103	20.0%	13	13.4%	90	21.6%	8.2%	
Q4	103	20.0%	22	22.7%	81	19.4%	−3.3%	
Q5 rich	103	20.0%	54	55.7%	49	11.8%	−43.9%	
	514		97		417			
Education								
Q1 least	103	20.0%	0	0.0%	103	24.7%	24.7%	
Q2	103	20.0%	11	11.3%	92	22.1%	10.7%	
Q3	103	20.0%	17	17.5%	86	20.6%	3.1%	
Q4	103	20.0%	29	29.9%	74	17.7%	−12.2%	
Q5 most	102	19.8%	40	41.2%	62	14.9%	−26.4%	
	514		97		417			
(b) Hypertension (%)								
All	n/a	33.3%	n/a	33.7%	n/a	33.2%	0.5%	
Males	n/a	30.4%	n/a	32.6%	n/a	29.9%	**2.7%**	*
Females	n/a	36.0%	n/a	34.6%	n/a	36.4%	**−1.8%**	*
Young adults	n/a	12.9%	n/a	12.4%	n/a	13.0%	−0.6%	
Adults	n/a	32.6%	n/a	34.0%	n/a	32.3%	**1.7%**	*
Older adults	n/a	63.2%	n/a	66.2%	n/a	62.5%	**3.7%**	*

Note: Q—Quintile, n—number, %—the proportion of column total, Urban—City, Rural—Regency. Data on district characteristics are from the World Bank, and hypertension data are from the Basic Health Survey 2018. For income, the grouping included 16.7–43.5% (quintile one), 12.5–16.6% (quintile two), 9.0–12.4% (quintile three), 6.0–8.9% (quintile four), 1.7–6.0% (quintile five). For education, the grouping included 8.6–53.1% (quintile one), 53.1–59.7% (quintile two), 59.9–64.8% (quintile three), 64.9–70.5% (quintile four), 70.6–86.4% (quintile five). Bold numbers with an asterisk (*) show statistical significance at 5% level (see Appendix B for the regression outputs).

**Table 3:** Geographic and socioeconomic disparity in hypertension among adults.

	All Districts (n = 514)						Urban (n = 97)						Rural (n = 417)					
				Young		Older				Young		Older				Young		Older
	All	Males	Females	Adults	Adults	Adults	All	Males	Females	Adults	Adults	Adults	All	Males	Females	Adults	Adults	Adults
	(1)	(2)	(3)	(4)	(5)	(6)	(7)	(8)	(9)	(10)	(11)	(12)	(13)	(14)	(15)	(16)	(17)	(18)
Region																		
Papua	26.3%	25.0%	27.5%	11.2%	26.3%	52.2%	28.3%	28.2%	28.4%	11.0%	29.4%	64.2%	26.0%	24.6%	27.4%	11.3%	26.0%	50.9%
Sulawesi	33.9%	30.8%	37.0%	13.0%	33.0%	65.6%	33.3%	33.2%	33.4%	12.4%	35.0%	63.6%	34.0%	30.5%	37.5%	13.2%	32.7%	65.9%
Kalimantan	40.1%	37.5%	42.8%	17.9%	40.7%	71.2%	38.4%	38.5%	38.3%	16.4%	40.0%	70.8%	40.4%	37.3%	43.7%	18.2%	40.8%	71.3%
Sumatera	30.7%	27.2%	34.1%	10.7%	29.8%	63.0%	30.2%	28.4%	31.4%	10.2%	29.8%	63.7%	30.8%	26.8%	34.8%	10.8%	29.8%	62.8%
Java	38.2%	35.1%	41.2%	14.5%	36.9%	66.3%	37.3%	36.0%	38.5%	13.9%	37.4%	68.6%	38.5%	34.7%	42.2%	14.7%	36.7%	65.5%
Absolute	**11.9%**	**10.1%**	**13.7%**	**3.2%**	**10.6%**	**14.2%**	**8.9%**	**7.9%**	**10.1%**	**2.9%**	**8.0%**	**4.5%**	**12.5%**	**10.1%**	**14.8%**	**3.4%**	**10.7%**	**14.6%**
Relative	**1.45**	**1.40**	**1.50**	**1.29**	**1.40**	**1.27**	**1.32**	**1.28**	**1.36**	**1.27**	**1.27**	**1.07**	**1.48**	**1.41**	**1.54**	**1.30**	**1.41**	**1.29**
Income																		
Q1 poor	27.9%	25.9%	29.9%	11.9%	27.6%	54.2%	31.0%	30.2%	31.7%	10.1%	32.2%	66.9%	27.8%	25.7%	29.8%	11.9%	27.4%	53.8%
Q2	32.7%	29.3%	36.0%	12.1%	31.5%	63.3%	31.8%	30.1%	33.6%	12.3%	31.4%	67.6%	32.7%	29.2%	36.1%	12.1%	31.6%	63.1%
Q3	35.7%	32.1%	39.2%	13.3%	34.5%	65.2%	31.8%	30.3%	33.4%	11.2%	32.5%	66.8%	36.2%	32.3%	40.1%	13.6%	34.8%	65.0%
Q4	34.5%	31.2%	37.8%	12.7%	33.7%	65.9%	33.0%	31.3%	34.5%	11.1%	33.1%	65.6%	34.9%	31.1%	38.7%	13.2%	33.8%	66.0%
Q5 rich	35.6%	33.7%	37.2%	14.4%	35.6%	67.0%	34.7%	34.1%	35.1%	13.4%	35.1%	66.1%	36.5%	33.4%	39.6%	15.5%	36.1%	68.1%
Absolute	7.7%	**7.9%**	7.4%	2.5%	8.0%	**12.9%**	3.7%	3.9%	3.4%	3.3%	2.9%	−0.8%	8.7%	7.6%	9.8%	3.6%	8.6%	**14.3%**
Relative	1.28	**1.30**	1.25	1.21	1.29	**1.24**	1.12	1.13	1.11	1.33	1.09	0.99	1.31	1.30	1.33	1.30	1.31	**1.27**
Education																		
Q1 least	32.6%	30.0%	35.3%	14.9%	32.2%	59.4%	n/a	n/a	n/a	n/a	n/a	n/a	32.6%	30.0%	35.3%	14.9%	32.2%	59.4%
Q2	33.6%	30.2%	37.0%	13.0%	33.1%	64.0%	35.2%	34.2%	36.2%	14.8%	36.1%	69.4%	33.4%	29.7%	37.1%	12.8%	32.7%	63.4%
Q3	33.6%	30.9%	36.4%	12.6%	32.9%	64.8%	34.0%	33.6%	34.3%	13.2%	34.5%	65.7%	33.5%	30.3%	36.8%	12.4%	32.6%	64.7%
Q4	32.8%	30.0%	35.5%	11.8%	32.1%	63.3%	33.0%	31.9%	34.0%	11.7%	33.4%	64.9%	32.7%	29.3%	36.1%	11.8%	31.6%	62.7%
Q5 most	33.7%	31.1%	36.1%	12.2%	32.6%	64.3%	33.7%	32.3%	34.6%	11.9%	33.7%	66.4%	33.7%	30.3%	37.0%	12.4%	31.9%	62.9%
Absolute	1.0%	1.1%	0.8%	**−2.7%**	0.4%	4.9%	−1.6%	−1.9%	−1.6%	−2.9%	−2.3%	−3.0%	1.1%	0.3%	1.8%	**−2.5%**	−0.3%	3.5%
Relative	1.03	1.04	1.02	**0.82**	1.01	1.08	0.96	0.94	0.96	0.81	0.94	0.96	1.03	1.01	1.05	**0.83**	0.99	1.06

Note: Q = Quintile; Java region includes Bali; Papua region includes Maluku and Nusa Tenggara. Income quintile used the district-level poverty rate (e.g., Q1 = 20% of districts with the highest poverty rate). Absolute (Relative)—Difference (Ratio) between Papua and Java as well as Q1 and Q5. For education, absolute (relative) was between Q1 and Q5 except among urban (Q2 and Q5). Boldface values show statistical significance at a 5l (see Appendix E for the regression outputs).

**Figure A1:**
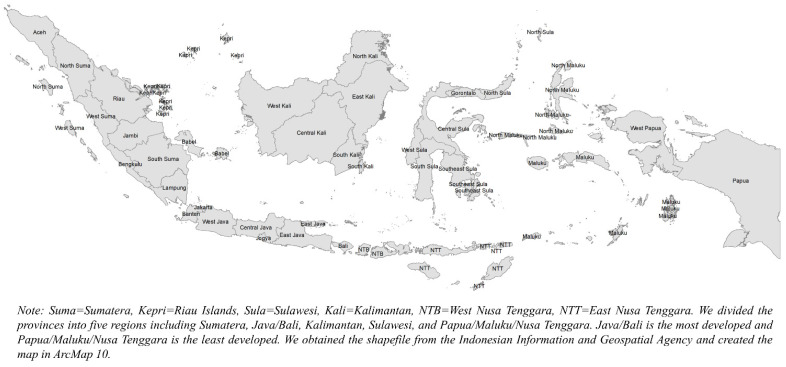
Map of Indonesia by province.

## Data Availability

Available from the authors upon reasonable request.

## Appendix B

**Table A1:** Regression outputs for urban/rural differences.

	All	Males	Females	Young Adults	Adults	Older Adults
	Coef	Coef	Coef	Coef	Coef	Coef
Rural	Reference					
Urban	0.52	2.70 **	−1.83 *	−0.57	1.77 *	3.68 **
Constant	33.17 **	29.92 **	36.39 **	13.00 **	32.26 **	62.49 **
Observations	514	514	514	514	514	514
R-squared	0.00	0.02	0.01	0.00	0.01	0.02

Note: Coef—OLS Coefficient; Significance level ** *p* < 0.01, * *p* < 0.05.

## Appendix C

**Table A2:** Ten districts with the lowest prevalence of hypertension among adults in Indonesia.

	Prevalence	Province	Region	Urban	Poverty	Education	Pop (000)
(a) All							
Kab. Nduga	9.7%	Papua	Papua	Rural	38%	9%	94
Kab. Tolikara	11.8%	Papua	Papua	Rural	33%	34%	131
Kab. Asmat	13.4%	Papua	Papua	Rural	27%	21%	88
Kab. Teluk Wondama	14.2%	West Papua	Papua	Rural	33%	39%	30
Kab. Yahukimo	14.4%	Papua	Papua	Rural	39%	12%	181
Kab. Lanny Jaya	14.4%	Papua	Papua	Rural	40%	46%	172
Kab. Mambramo Raya	15.7%	Papua	Papua	Rural	30%	51%	21
Kab. Sorong Selatan	16.3%	West Papua	Papua	Rural	19%	56%	43
Kab. Jayawijaya	16.4%	Papua	Papua	Rural	39%	67%	206
Kab. Mambramo Tengah	16.9%	Papua	Papua	Rural	37%	54%	46
Average					34%	39%	101
(b) Males							
Kab. Nduga	11%	Papua	Papua	Rural	38%	9%	94
Kab. Buton Tengah	12%	Southeast Sulawesi	Sulawesi	Rural	15%	80%	89
Kab. Tolikara	13%	Papua	Papua	Rural	33%	34%	131
Kab. Teluk Wondama	14%	West Papua	Papua	Rural	33%	39%	30
Kab. Sorong Selatan	14%	West Papua	Papua	Rural	19%	56%	43
Kab. Lanny Jaya	14%	Papua	Papua	Rural	40%	46%	172
Kab. Keerom	14%	Papua	Papua	Rural	17%	61%	54
Kab. Asmat	15.0%	Papua	Papua	Rural	27%	21%	88
Kab. Intan Jaya	15.0%	Papua	Papua	Rural	43%	9%	46
Kab. Padang Lawas	15.2%	North Sumatera	Sumatera	Rural	8%	63%	257
Average					27%	42%	100
(c) Females							
Kab. Nduga	8%	Papua	Papua	Rural	38%	9%	94
Kab. Tolikara	11%	Papua	Papua	Rural	33%	34%	131
Kab. Yahukimo	12%	Papua	Papua	Rural	39%	12%	181
Kab. Asmat	12%	Papua	Papua	Rural	27%	21%	88
Kab. Jayawijaya	12.5%	Papua	Papua	Rural	39%	67%	206
Kab. Mambramo Tengah	13.8%	Papua	Papua	Rural	37%	54%	46
Kab. Teluk Wondama	15.1%	West Papua	Papua	Rural	33%	39%	30
Kab. Lanny Jaya	15.2%	Papua	Papua	Rural	40%	46%	172
Kab. Tambrauw	15.7%	West Papua	Papua	Rural	35%	47%	14
Kab. Mambramo Raya	16.0%	Papua	Papua	Rural	30%	51%	21
Average					35%	38%	98
(d) Young adults							
Kab. Buton Tengah	1%	Southeast Sulawesi	Sulawesi	Rural	15%	80%	89
Kab. Kep. Mentawai	1%	West Sumatera	Sumatera	Rural	14%	40%	85
Kab. Padang Lawas	2%	North Sumatera	Sumatera	Rural	8%	63%	257
Kab. Halmahera Tengah	3%	North Maluku	Papua	Rural	14%	63%	50
Kab. Sarolangun Bangko	3%	Jambi	Sumatera	Rural	9%	59%	278
Kab Pringsewu	3%	Lampung	Sumatera	Rural	11%	63%	387
Kab. Dompu	3%	West Nusa Tenggara	Papua	Rural	12%	70%	238
Kab. Biak Numfor	3%	Papua	Papua	Rural	26%	62%	139
Kab. Nias Utara	4%	North Sumatera	Sumatera	Rural	27%	73%	134
Kab. Bengkulu Selatan	4%	Bengkulu	Sumatera	Rural	19%	64%	152
Average					15%	64%	181
(e) Adults							
Kab. Nduga	9.8%	Papua	Papua	Rural	38%	9%	94
Kab. Tolikara	10.6%	Papua	Papua	Rural	33%	34%	131
Kab. Mambramo Raya	11.8%	Papua	Papua	Rural	30%	51%	21
Kab. Asmat	13.6%	Papua	Papua	Rural	27%	21%	88
Kab. Buton Tengah	14.7%	Southeast Sulawesi	Sulawesi	Rural	15%	80%	89
Kab. Yahukimo	14.7%	Papua	Papua	Rural	39%	12%	181
Kab. Teluk Wondama	14.8%	West Papua	Papua	Rural	33%	39%	30
Kab. Lanny Jaya	15.1%	Papua	Papua	Rural	40%	46%	172
Kab. Jayawijaya	15.4%	Papua	Papua	Rural	39%	67%	206
Kab. Padang Lawas	15.9%	North Sumatera	Sumatera	Rural	8%	63%	257
Average					30%	42%	127
(f) Older adults							
Kab. Yahukimo	0.0%	Papua	Papua	Rural	39%	12%	181
Kab. Pegunungan Bintang	0.0%	Papua	Papua	Rural	31%	21%	72
Kab. Nduga	0.0%	Papua	Papua	Rural	38%	9%	94
Kab. Tapanuli Selatan	6.3%	North Sumatera	Sumatera	Rural	9%	68%	275
Kab. Jayawijaya	17.7%	Papua	Papua	Rural	39%	67%	206
Kab. Mambramo Tengah	18.0%	Papua	Papua	Rural	37%	54%	46
Kab. Asmat	26.4%	Papua	Papua	Rural	27%	21%	88
Kab. Peg Arfak	29.2%	West Papua	Papua	Rural	36%	48%	28
Kab. Paniayi	32.2%	Papua	Papua	Rural	37%	25%	164
Kab. Lanny Jaya	32.9%	Papua	Papua	Rural	40%	46%	172
Average					33%	37%	133

Note: Urban—City, Rural—Regency; Pop—Population. The districts are ordered by prevalence (column 1).

## Appendix D

**Table A3:** Ten districts with the highest prevalence of hypertension among adults in Indonesia, 2018.

		Prevalence	Province	Region	Urban	Poverty	Education	Pop (000)
(a) All								
Kab. Hulu Sungai Tengah		53.2%	South Kalimantan	Kalimantan	Rural	6%	66%	260
Kab. Tabalong		50.8%	South Kalimantan	Kalimantan	Rural	6%	61%	239
Kab. Ciamis		50.5%	West Java	Jawa	Rural	7%	51%	1168
Kab. Kutai Barat		49.8%	East Kalimantan	Kalimantan	Rural	9%	60%	146
Kota Banjarmasin		48.9%	South Kalimantan	Kalimantan	Urban	4%	55%	675
Kab. Cianjur		48.7%	West Java	Jawa	Rural	10%	45%	2243
Kab. Kuningan		48.5%	West Java	Jawa	Rural	12%	67%	1055
Kota Madiun		48.2%	East Java	Jawa	Urban	4%	80%	175
Kab. Barito Kuala		47.6%	South Kalimantan	Kalimantan	Rural	5%	62%	298
Kota Tomohon		47.2%	North Sulawesi	Sulawesi	Urban	6%	71%	100
Average						7%	62%	636
(b) Males								
Kab. Kutai Barat		51.1%	East Kalimantan	Kalimantan	Rural	9%	60%	146
Kab. Tabalong		49.9%	South Kalimantan	Kalimantan	Rural	6%	61%	239
Kota Madiun		49.7%	East Java	Jawa	Urban	4%	80%	175
Kab. Hulu Sungai Tengah		49.5%	South Kalimantan	Kalimantan	Rural	6%	66%	260
Kota Banjarmasin		48.8%	South Kalimantan	Kalimantan	Urban	4%	55%	675
Kota Tomohon		48.6%	North Sulawesi	Sulawesi	Urban	6%	71%	100
Kota Singkawang		47.9%	West Kalimantan	Kalimantan	Urban	5%	60%	207
Kab. Karo		47.4%	North Sumatera	Sumatera	Rural	9%	74%	389
Kab. Barito Kuala		46.0%	South Kalimantan	Kalimantan	Rural	5%	62%	298
Kab. Kutai Kartanegara		44.3%	East Kalimantan	Kalimantan	Rural	7%	74%	716
Average						6%	66%	321
(c) Females								
Kab. Ciamis		57.2%	West Java	Jawa	Rural	7%	51%	1168
Kab. Hulu Sungai Tengah		56.7%	South Kalimantan	Kalimantan	Rural	6%	66%	260
Kab. Cianjur		53.6%	West Java	Jawa	Rural	10%	45%	2243
Kab. Kuningan		53.3%	West Java	Jawa	Rural	12%	67%	1055
Melawi		53.3%	West Kalimantan	Kalimantan	Rural	13%	41%	196
Kab. Garut		52.8%	West Java	Jawa	Rural	9%	51%	2547
Kab. Anambas Kep		52.1%	Riau Islands	Sumatera	Rural	7%	77%	40
Kab. Tanah Laut		52.0%	South Kalimantan	Kalimantan	Rural	4%	55%	324
Kab. Nganjuk		51.9%	East Java	Jawa	Rural	12%	63%	1041
Kota Sukabumi		51.8%	West Java	Jawa	Urban	7%	73%	318
Average						9%	59%	919
(d) Young adults								
Kab. Pegunungan Bintang		37.6%	Papua	Papmalnus	Rural	31%	21%	72
Kab. Tabalong		33.3%	South Kalimantan	Kalimantan	Rural	6%	61%	239
Kab. Mahakam Ulu		30.5%	East Kalimantan	Kalimantan	Rural	12%	52%	26
Kab. Hulu Sungai Tengah		28.8%	South Kalimantan	Kalimantan	Rural	6%	66%	260
Kab. Peg Arfak		27.6%	West Papua	Papmalnus	Rural	36%	48%	28
Melawi		26.7%	West Kalimantan	Kalimantan	Rural	13%	41%	196
Kab. Brebes		26.5%	Central Java	Jawa	Rural	17%	50%	1781
Kab. Karo		26.2%	North Sumatera	Sumatera	Rural	9%	74%	389
Kab. Kutai Kartanegara		26.1%	East Kalimantan	Kalimantan	Rural	7%	74%	716
Kota Cimahi		25.9%	West Java	Jawa	Urban	5%	72%	586
Average						14%	56%	429
(e) Adults								
Kab. Kutai Barat		52.8%	East Kalimantan	Kalimantan	Rural	9%	60%	146
Kab. Hulu Sungai Tengah		52.1%	South Kalimantan	Kalimantan	Rural	6%	66%	260
Kota Banjarmasin		50.9%	South Kalimantan	Kalimantan	Urban	4%	55%	675
Kab. Tabalong		50.7%	South Kalimantan	Kalimantan	Rural	6%	61%	239
Kota Madiun		49.4%	East Java	Jawa	Urban	4%	80%	175
Kota Sukabumi		48.9%	West Java	Jawa	Urban	7%	73%	318
Kota Tomohon		48.7%	North Sulawesi	Sulawesi	Urban	6%	71%	100
Melawi		48.2%	West Kalimantan	Kalimantan	Rural	13%	41%	196
Kab. Kutai Kartanegara		48.1%	East Kalimantan	Kalimantan	Rural	7%	74%	716
Kab. Cianjur		47.7%	West Java	Jawa	Rural	10%	45%	2243
Average						7%	63%	507
(f) Older adults								
Kab. Diyai		100.0%	Papua	Papmalnus	Rural	43%	51%	69
Kab. Berau		86.2%	East Kalimantan	Kalimantan	Rural	5%	71%	208
Kab. Buton Selatan		85.9%	Southeast Sulawesi	Sulawesi	Rural	15%	44%	77
Kab. Barito Kuala		84.7%	South Kalimantan	Kalimantan	Rural	5%	62%	298
Kab. Hulu Sungai Tengah		82.9%	South Kalimantan	Kalimantan	Rural	6%	66%	260
Kab Belitung Timur		82.8%	Bangka Belitung	Sumatera	Rural	7%	62%	119
Kab. PPU		82.3%	East Kalimantan	Kalimantan	Rural	7%	69%	154
Kota Banjarmasin		82.2%	South Kalimantan	Kalimantan	Urban	4%	55%	675
Kab. Belitung		81.9%	Bangka Belitung	Sumatera	Rural	8%	51%	175
Kab. Anambas Kep		81.6%	Riau Islands	Sumatera	Rural	7%	77%	40
Average						11%	61%	208

Note: Urban—City, Rural—Regency; Pop—Population. The districts are ordered by prevalence (column 1).

## Appendix E

**Table A4:** Regression outputs for geographic and socioeconomic disparity in hypertension.

	All	Males	Females	Young Adults	Adults	Older Adults
	Coef	Coef	Coef	Coef	Coef	Coef
(a) All districts (N = 514)						
Papua	Reference					
Java	10.74 **	8.72 **	12.82 **	3.68 **	9.43 **	11.01 **
Sumatera	3.31 **	0.87	5.72 **	0.23	2.50 **	7.72 **
Kalimantan	12.94 **	11.07 **	15.22 **	6.75 **	13.25 **	15.80 **
Sulawesi	6.69 **	5.00 **	8.37 **	2.45 **	5.81 **	10.45 **
Income						
Quintile 1 poor	Reference					
Quintile 2	1.93 *	1.35	2.48 **	−0.09	1.52	4.35 **
Quintile 3	2.74 **	2.23 *	3.17 **	0.06	2.39 **	4.57 **
Quintile 4	1.86 *	1.69	2.00 *	−0.42	1.79 *	5.21 **
Quintile 5 rich	1.07	2.18 *	−0.40	0.23	1.62	4.70 **
Education						
Quintile 1 least	Reference					
Quintile 2	−0.35	−0.74	0.19	−1.93 **	−0.21	1.87
Quintile 3	0.32	0.63	0.14	−2.08 **	0.30	3.29 *
Quintile 4	−0.31	−0.03	−0.58	−2.72 **	−0.40	1.89
Quintile 5 most	0.52	1.20	−0.21	−1.99 **	0.24	2.29
(b) Urban (N = 97)						
Papua	Reference					
Java	10.77 **	9.66 **	12.16 **	3.43 *	10.39 **	7.07 **
Sumatera	2.26	0.74	3.36	−0.54	0.96	0.06
Kalimantan	12.29 **	12.21 **	12.63 **	5.64 **	13.38 **	9.74 **
Sulawesi	7.40 **	7.56 **	7.47 **	1.88	8.60 **	1.76
Income						
Quintile 1 poor	Reference					
Quintile 2	−2.05	−2.71	−1.17	0.75	−3.72	−2.30
Quintile 3	−0.57	−1.23	0.31	0.51	−1.04	−1.07
Quintile 4	−4.44	−5.38	−3.70	−1.79	−6.15 *	−6.08
Quintile 5 rich	−3.01	−3.10	−3.46	−0.02	−4.54	−6.30
Education						
Quintile 1 least	n/a	n/a	n/a	n/a	n/a	n/a
Quintile 2	Reference					
Quintile 3	−0.10	0.93	−1.07	−1.10	−0.06	−4.02
Quintile 4	−0.69	−0.26	−1.03	−2.05	−0.69	−4.37
Quintile 5 most	1.91	2.26	1.29	−1.00	2.00	−1.55
(c) Rural (N = 417)						
Papua	Reference					
Java	10.97 **	8.90**	13.01 **	3.77 **	9.69 **	12.04 **
Sumatera	3.42 **	1.02	5.89 **	0.28	2.99 **	9.22 **
Kalimantan	13.02 **	11.58 **	14.90 **	6.84 **	13.89 **	16.96 **
Sulawesi	6.67 **	4.86 **	8.47 **	2.55 **	5.73 **	12.21 **
Income						
Quintile 1 poor	Reference					
Quintile 2	2.03 *	1.50	2.51 **	−0.18	1.66	4.00 *
Quintile 3	2.73 **	2.04 *	3.32 **	−0.04	2.12 *	3.71 *
Quintile 4	2.51 **	1.98 *	3.00 **	−0.11	2.06 *	5.18 **
Quintile 5 rich	1.33	1.12	1.20	0.34	0.97	4.96 *
Education						
Quintile 1 least	Reference					
Quintile 2	−0.39	−0.86	0.21	−2.02 **	−0.29	1.35
Quintile 3	0.56	0.57	0.65	−2.02 **	0.37	3.28 *
Quintile 4	0.08	−0.13	0.21	−2.48 **	−0.42	1.66
Quintile 5 most	0.24	0.55	−0.04	−1.84 *	−0.66	0.42

Note: Coef—OLS Coefficient; Significance level ** *p* < 0.01, * *p* < 0.05.

## References

[1] WHO (2021). Hypertension Fact Sheets. https://www.who.int/news-room/fact-sheets/detail/hypertension.

[2] GBD 2019 Risk Factors Collaborators (2020). Global burden of 87 risk factors in 204 countries and territories, 1990–2019: A systematic analysis for the Global Burden of Disease Study 2019. Lancet.

[3] GBD 2019 Diseases and Injuries Collaborators (2020). Global burden of 369 diseases and injuries in 204 countries and territories, 1990–2019: A systematic analysis for the Global Burden of Disease Study 2019. Lancet.

[4] Meyers S., Earnshaw V., D’Ambrosio B., Courchesne N., Werb D., Smith L. (2021). The intersection of gender and drug use-related stigma: A mixed methods systematic review and synthesis of the literature. Drug Alcohol. Depend.

[5] NIHRD (2018). Report of Riskesdas.

[6] Mboi N., Surbakti I.M., Trihandini I., Elyazar I., Smith K.H., Ali P.B., Kosen S., Flemons K., Ray S.E., Cao J. (2018). On the road to universal health care in Indonesia, 1990–2016: A systematic analysis for the Global Burden of Disease Study 2016. Lancet.

[7] Busingye D., Arabshahi S., Subasinghe A.K., Evans R.G., Riddell M.A., Thrift A.G. (2014). Do the socioeconomic and hypertension gradients in rural populations of low- and middle-income countries differ by geographical region? A systematic review and meta-analysis. Int. J. Epidemiol..

[8] Mishra S.R., Ghimire S., Shrestha N., Shrestha A., Virani S.S. (2019). Socio-economic inequalities in hypertension burden and cascade of services: Nationwide cross-sectional study in Nepal. J. Hum. Hypertens.

[9] Kershaw K.N., Roux A.V.D., Carnethon M., Darwin C., Goff D.C., Post W., Schreiner P.J., Watson K. (2010). Geographic variation in hypertension prevalence among blacks and whites: The multi-ethnic study of atherosclerosis. Am. J. Hypertens.

[10] Morenoff J.D., House J.S., Hansen B.B., Williams D.R., Kaplan G.A., Hunte H.E. (2007). Understanding social disparities in hypertension prevalence, awareness, treatment, and control: The role of neighborhood context. Soc. Sci. Med..

[11] Cho K.H., Lee S.G., Nam C.M., Lee E.J., Jang S.-Y., Lee S.-H., Park E.-C. (2016). Disparities in socioeconomic status and neighborhood characteristics affect all-cause mortality in patients with newly diagnosed hypertension in Korea: A nationwide cohort study, 2002-2013. Int. J. Equity Health.

[12] Li Y., Wang L., Feng X., Zhang M., Huang Z., Deng Q., Zhou M., Astell-Burt T., Wang L. (2018). Geographical variations in hypertension prevalence, awareness, treatment and control in China: Findings from a nationwide and provincially representative survey. J. Hypertens.

[13] Yin M., Augustin B., Fu Z., Yan M., Fu A., Yin P. (2016). Geographic Distributions in Hypertension Diagnosis, Measurement, Prevalence, Awareness, Treatment and Control Rates among Middle-aged and Older Adults in China. Sci. Rep..

[14] Laohasiriwong W., Puttanapong N., Singsalasang A. (2018). Prevalence of hypertension in Thailand: Hotspot clustering detected by spatial analysis. Geospat. Health.

[15] Ayuningtyas D., Hapsari D., Rachmalina R., Amir V., Rachmawati R., Kusuma D. (2022). Geographic and Socioeconomic Disparity in Child Undernutrition across 514 Districts in Indonesia. Nutrients.

[16] Hapsari D., Nainggolan O., Kusuma D. (2020). Hotspots and Regional Variation in Smoking Prevalence Among 514 Districts in Indonesia: Analysis of Basic Health Research 2018. Glob. J. Health Sci..

[17] Bella A., Akbar M., Kusnadi G., Herlinda O., Regita P., Kusuma D. (2021). Socioeconomic and Behavioral Correlates of COVID-19 Infections among Hospital Workers in the Greater Jakarta Area, Indonesia: A Cross-Sectional Study. Int. J. Environ. Res. Public. Health.

[18] Di Cesare M., Khang Y.-H., Asaria P., Blakely T., Cowan M.J., Farzadfar F., Guerrero R., Ikeda N., Kyobutungi C., Msyamboza K.P. (2013). Inequalities in non-communicable diseases and effective responses. Lancet.

[19] Zhou B., Carrillo-Larco R.M., Danaei G., Riley L.M., Paciorek C.J., Stevens G.A., Gregg E.W., Bennett J.E., Solomon B., Singleton R.K. (2021). Worldwide trends in hypertension prevalence and progress in treatment and control from 1990 to 2019: A pooled analysis of 1201 population-representative studies with 104 million participants. Lancet.

[20] Daştan İ., Erem A., Çetinkaya V. (2017). Urban and rural differences in hypertension risk factors in Turkey. Anatol. J. Cardiol..

[21] Appiah F., Ameyaw E.K., Oduro J.K., Baatiema L., Sambah F., Seidu A.-A., Ahinkorah B.O., Budu E. (2021). Rural-urban variation in hypertension among women in Ghana: Insights from a national survey. BMC Public Health.

[22] Atanasova P., Kusuma D., Pineda E., Anjana R.M., De Silva L., Hanif A.A., Hasan M., Hossain M., Indrawansa S., Jayamanne D. (2022). Food environments and obesity: A geospatial analysis of the South Asia Biobank, income and sex inequalities. SSM-Popul. Heal..

[23] Kusuma D., Atanasova P., Pineda E., Anjana R.M., De Silva L., Hanif A.A., Hasan M., Hossain M., Indrawansa S., Jayamanne D. (2022). Food environment and diabetes mellitus in South Asia: A geospatial analysis of health outcome data. PLOS Med..

[24] AlQurashi A., Kusuma D., AlJishi H., AlFaiz A., AlSaad A. (2021). Density of Fast Food Outlets around Educational Facilities in Riyadh, Saudi Arabia: Geospatial Analysis. Int. J. Environ. Res. Public Health.

[25] Sivasampu S., Teh X.R., Lim Y.M.F., Ong S.M., Ang S.H., Husin M., Khamis N., Jaafar F.S.A., Wong W.J., Shanmugam S. (2020). Study protocol on Enhanced Primary Healthcare (EnPHC) interventions: A quasi-experimental controlled study on diabetes and hypertension management in primary healthcare clinics. Prim. Health Care Res. Dev..

[26] Song P., Gupta A., Goon I.Y., Hasan M., Mahmood S., Pradeepa R., Siddiqui S., Frost G.S., Kusuma D., Miraldo M. Data resource profile: Understanding the patterns and determinants of health in South Asians-the South Asia Biobank. Int. J. Epidemiol..

[27] Kusuma D. (2021). Lessons from primary health care in the United Kingdom. J. Adm. Kesehat. Indones.

[28] Puspikawati S.I., Dewi D.M.S.K., Astutik E., Kusuma D., Melaniani S., Sebayang S.K. (2021). Density of outdoor food and beverage advertising around gathering place for children and adolescent in East Java, Indonesia. Public Health Nutr..

[29] Ahsan A., Wiyono N.H., Veruswati M., Adani N., Kusuma D., Amalia N. (2020). Comparison of tobacco import and tobacco control in five countries: Lessons learned for Indonesia. Glob. Health.

[30] Handayani S., Rachmani E., Saptorini K., Manglapy Y., Nurjanah, Ahsan A., Kusuma D. (2021). Is Youth Smoking Related to the Density and Proximity of Outdoor Tobacco Advertising Near Schools? Evidence from Indonesia. Int. J. Environ. Res. Public Health Artic. Public Health.

[31] Sebayang S.K., Dewi D.M.S.K., Puspikawati S.I., Astutik E., Melaniani S., Kusuma D. (2021). Spatial analysis of outdoor tobacco advertisement around children and adolescents in Indonesia. Glob. Public Health.

[32] Adisasmito W., Amir V., Atin A., Megraini A., Kusuma D. (2020). Density of cigarette retailers around educational facilities in Indonesia. Int. J. Tuberc. Lung Dis..

[33] Nurjanah N., Manglapy Y.M., Handayani S., Ahsan A., Sutomo R., Dewi F.S.T., Chang P., Kusuma D. (2020). Density of tobacco advertising around schools. Int. J. Tuberc. Lung Dis..

[34] Ayuningtyas D., Kusuma D., Amir V., Tjandrarini D.H., Andarwati P. (2022). Disparities in Obesity Rates among Adults: Analysis of 514 Districts in Indonesia. Nutrients.

[35] Drobniewski F., Kusuma D., Broda A., Castro-Sánchez E., Ahmad R. (2022). COVID-19 Vaccine Hesitancy in Diverse Groups in the UK—Is the Driver Economic or Cultural in Student Populations. Vaccines.

